# HIV‐Associated Diffuse Large B‐Cell Lymphoma Treated With Outpatient Pola‐R‐CHP Without Antiretroviral Therapy Modification: A Case Report

**DOI:** 10.1155/crdi/7583461

**Published:** 2026-08-10

**Authors:** Yoshikazu Ikoma, Masashi Ishihara, Hitomi Sugiyama, Daisuke Okamoto, Naoki Hayase, Kimihiro Yamaguchi, Takuro Matsumoto, Nobuyuki Tetsuka, Nobuhiko Nakamura, Hiroshi Nakamura, Nobuhiro Kanemura, Masahito Shimizu, Hisashi Tsurumi

**Affiliations:** ^1^ Department of Hematology and Infectious Disease, Gifu University Hospital, Gifu, Japan, gifu-u.ac.jp; ^2^ Center for Nutrition Support and Infection Control, Gifu University Hospital, Gifu, Japan, gifu-u.ac.jp; ^3^ AIDS Clinical Center, Gifu University Hospital, Gifu, Japan, gifu-u.ac.jp; ^4^ Department of Pharmacy, Gifu University Hospital, Gifu, Japan, gifu-u.ac.jp; ^5^ Nursing Department, Gifu University Hospital, Gifu, Japan, gifu-u.ac.jp; ^6^ Department of Hematology, Gifu Municipal Hospital, Gifu, Japan, gmhosp.jp; ^7^ Department of Infection Control, Gifu University Graduate School of Medicine, Gifu, Japan, gifu-u.ac.jp; ^8^ Department of Internal Medicine, Matsunami General Hospital, Gifu, Japan, matsunami-hsp.or.jp

**Keywords:** case report, diffuse large B-cell lymphoma, HIV-associated lymphoma, Pola-R-CHP, real-world evidence

## Abstract

**Introduction:**

People living with HIV (PLWH) remain at increased risk of diffuse large B‐cell lymphoma (DLBCL). Polatuzumab vedotin plus rituximab, cyclophosphamide, doxorubicin, and prednisone (Pola‐R‐CHP) has emerged as a standard first‐line option for DLBCL in the general population, but the pivotal POLARIX trial excluded PLWH, leaving limited evidence on feasibility with contemporary antiretroviral therapy (ART).

**Case Presentation:**

A 61‐year‐old man presented with progressive anorexia and weight loss. With durable virologic suppression (plasma HIV‐1 RNA below 20 copies/mL) and immune reconstitution (CD4+ T‐cell count approximately 300 cells/µL) on bictegravir/emtricitabine/tenofovir alafenamide (BIC/FTC/TAF), he was diagnosed with nongerminal center B‐cell DLBCL (Lugano stage II; International Prognostic Index score 2). Pola‐R‐CHP was administered every 21 days for six cycles (Cycle 1: inpatient and Cycles 2–6: outpatient), followed by two additional rituximab cycles, without ART modification. No grade 3–4 nonhematologic toxicity, febrile neutropenia, or serious infections occurred. HIV‐1 RNA remained below 20 copies/mL throughout treatment, and CD4+ T‐cell counts showed no clinically meaningful decline. End‐of‐treatment fluorodeoxyglucose positron emission tomography/computed tomography (FDG‐PET–CT) demonstrated a partial metabolic response with two small residual FDG‐avid foci (∼1 cm; SUVmax ∼4‐5). At the last follow‐up, the patient had no clinical or radiologic evidence of progression.

**Conclusion:**

Outpatient‐delivered Pola‐R‐CHP appeared feasible in PLWH with virologic suppression and immune reconstitution receiving BIC/FTC/TAF, without virologic breakthrough or unexpected toxicity. Prospective inclusion of PLWH in polatuzumab‐containing frontline studies is warranted.

## 1. Introduction

People living with HIV (PLWH) remain at increased risk of aggressive B‐cell lymphomas despite advances in antiretroviral therapy (ART), and diffuse large B‐cell lymphoma (DLBCL) continues to represent a major clinical challenge in this setting [1, 2]. With contemporary ART, many individuals achieve durable virologic suppression and immune reconstitution, enabling delivery of curative‐intent chemoimmunotherapy; however, the optimal first‐line regimen for HIV‐associated DLBCL (HIV‐DLBCL) remains debated, and evidence continues to rely largely on heterogeneous, nonrandomized data [1, 2].

Rituximab‐containing regimens such as rituximab plus cyclophosphamide, doxorubicin, vincristine, and prednisone (R‐CHOP) and etoposide, prednisone, vincristine, cyclophosphamide, doxorubicin, and rituximab (EPOCH‐R) are commonly used for HIV‐DLBCL. Although some contemporary guidelines and expert reviews favor EPOCH‐R for selected patients, high‐quality head‐to‐head comparisons with R‐CHOP in PLWH are limited; therefore, regimen selection in routine practice often depends on institutional experience, available resources, and patient‐specific considerations, including feasibility and patient preferences, particularly because delivery of EPOCH‐R may require central venous access, continuous infusions, and, depending on the setting, either substantial ambulatory support infrastructure or inpatient management [1–3].

Polatuzumab vedotin plus rituximab, cyclophosphamide, doxorubicin, and prednisone (Pola‐R‐CHP) has been established as a standard first‐line option for DLBCL in the general population [4]. Notably, the pivotal POLARIX trial excluded individuals with HIV infection, leaving uncertainty regarding the safety and effectiveness of polatuzumab‐containing frontline therapy in PLWH [4]. More broadly, PLWH have often been excluded from pivotal trials of novel cancer agents, contributing to persistent evidence gaps and reinforcing the value of carefully documented real‐world experiences [5].

Here, we describe an individual with HIV‐DLBCL and preserved immune function who completed 6 cycles of Pola‐R‐CHP followed by 2 additional rituximab cycles while maintaining bictegravir/emtricitabine/tenofovir alafenamide (BIC/FTC/TAF), without virologic breakthrough or unexpected toxicity.

## 2. Case Presentation

**FIGURE 1:**
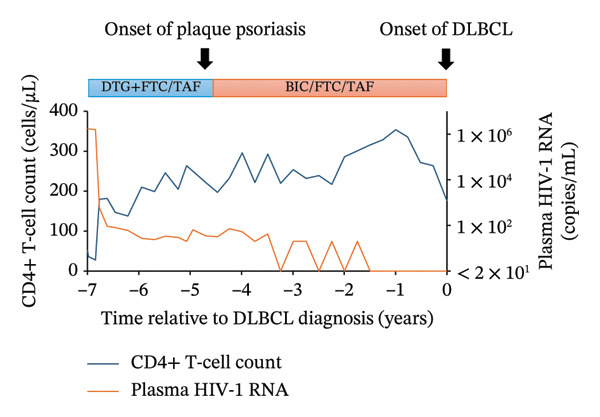
Longitudinal changes in CD4+ T‐cell count and plasma HIV‐1 RNA levels from HIV diagnosis to the onset of DLBCL. The left *y*‐axis indicates the CD4+ T‐cell count (cells/µL), and the right *y*‐axis indicates plasma HIV‐1 RNA levels (copies/mL) plotted on a logarithmic scale. Time is shown relative to the diagnosis of DLBCL (year 0). The onset of plaque psoriasis and DLBCL are indicated by arrows. DLBCL, diffuse large B‐cell lymphoma; DTG, dolutegravir; BIC, bictegravir; FTC, emtricitabine; TAF, tenofovir alafenamide.

**FIGURE 2:**
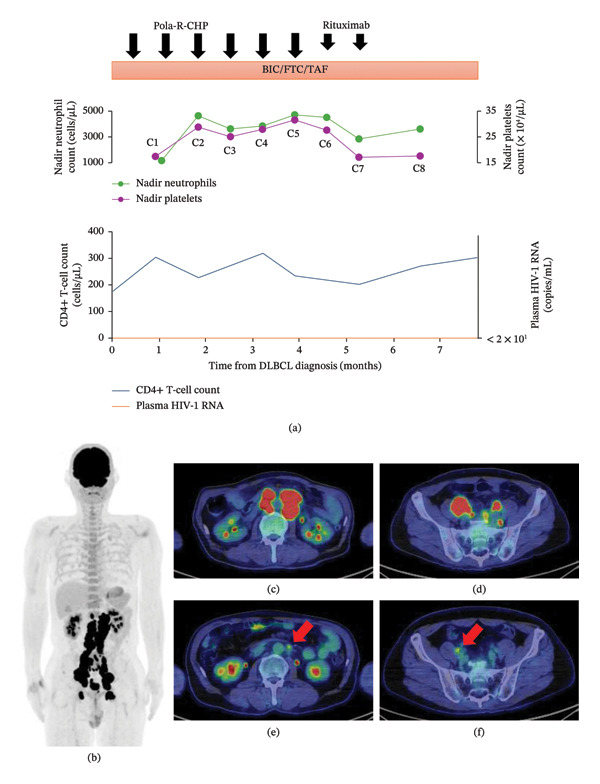
Clinical course, antiretroviral therapy, and metabolic response to Pola‐R‐CHP in an HIV‐associated DLBCL. (a) The upper panel shows nadir neutrophil counts (green line, left *y*‐axis) and nadir platelet counts (purple line, right *y*‐axis) for each cycle of Pola‐R‐CHP; C1–C8 denote cycles 1–8, respectively. The lower panel shows longitudinal CD4+ T‐cell counts (blue line, left *y*‐axis) and plasma HIV‐1 RNA levels (orange line, right *y*‐axis) from the time of DLBCL diagnosis through follow‐up. The upper bar indicates continuous BIC/FTC/TAF therapy. Black arrows denote each cycle of Pola‐R‐CHP, followed by additional rituximab infusions. Plasma HIV‐1 RNA remained below 20 copies/mL throughout the course, and hematologic toxicity remained manageable without febrile neutropenia or serious infection. (b) Baseline maximum‐intensity projection PET image at DLBCL diagnosis showing widespread FDG‐avid lymphadenopathy in the para‐aortic and pelvic regions. (c, d) Axial PET–CT images at baseline demonstrating bulky para‐aortic (c) and bilateral external iliac (d) lymph node involvement with intense FDG uptake (maximum standardized uptake value [SUVmax] 40.0 in the dominant para‐aortic mass). (e, f) Axial PET–CT images obtained at the end of Pola‐R‐CHP treatment demonstrate a marked reduction in both size and FDG uptake of the para‐aortic (e) and right external iliac (f) lymph nodes (red arrows). Two small residual FDG‐avid foci remained, each measuring approximately 1 cm in diameter, with SUVmax of 4.2 in (e) and 4.9 in (f). These residual lesions were substantially smaller and less metabolically active than at baseline, consistent with a partial metabolic response according to the Lugano criteria. DLBCL, diffuse large B‐cell lymphoma; C, cycle; BIC, bictegravir; FTC, emtricitabine; TAF, tenofovir alafenamide; Pola‐R‐CHP, polatuzumab vedotin plus rituximab, cyclophosphamide, doxorubicin, and prednisone; PET, positron emission tomography; CT, computed tomography; FDG, fluorodeoxyglucose; SUVmax, maximum standardized uptake value.

A 61‐year‐old man with a 7‐year history of HIV infection was referred to our hospital with progressive anorexia and unintentional weight loss. He had been diagnosed with AIDS at the time of HIV diagnosis, when his CD4+ T‐cell count was 28 cells/µL and plasma HIV‐1 RNA was 1.7 × 10^6^ copies/mL (Figure 1). Initial ART with dolutegravir plus emtricitabine/tenofovir alafenamide (DTG + FTC/TAF) achieved virologic suppression, and his regimen was later switched to BIC/FTC/TAF for convenience. Approximately 2 years after initiation of ART, he developed plaque psoriasis, which was managed with topical corticosteroids and apremilast. His medical history was otherwise unremarkable. His family history was noncontributory. At the time of lymphoma diagnosis, his plasma HIV‐1 RNA remained below 20 copies/mL, and his CD4+ T‐cell count had recovered to approximately 300 cells/µL, indicating sustained immune reconstitution. Longitudinal CD4+ T‐cell counts and plasma HIV‐1 RNA levels from lymphoma diagnosis through follow‐up are shown in Figure 2(a).

Contrast‐enhanced CT revealed bulky para‐aortic lymphadenopathy, and PET–CT demonstrated intense FDG uptake in the dominant mass, with a maximum standardized uptake value (SUVmax) of 40.0 (Figures 2(b), 2(c), and 2(d)). Endoscopic ultrasound‐guided needle biopsy of the para‐aortic node confirmed DLBCL, not otherwise specified, nongerminal center B‐cell (non‐GCB) subtype [6, 7]. Fluorodeoxyglucose positron emission tomography/computed tomography (FDG‐PET–CT) staging evaluation indicated Lugano stage II [8]. The patient had an International Prognostic Index (IPI) score of 2 (low–intermediate risk) [9] and an Eastern Cooperative Oncology Group performance status (ECOG PS) of 0 [10]. Laboratory tests showed elevated lactate dehydrogenase (285 U/L) and soluble interleukin‐2 receptor (4030 U/mL).

Given his preserved performance status and immune function, standard inpatient chemoimmunotherapy with EPOCH‐R, as recommended in contemporary clinical practice guidelines for HIV‐DLBCL [3], was initially considered appropriate. However, the patient strongly wished to maintain his social activities and avoid prolonged hospitalization. Considering recent data establishing Pola‐R‐CHP as a standard first‐line option for DLBCL, we therefore proposed an outpatient‐based Pola‐R‐CHP regimen as an alternative, and the patient preferred this approach. The limited evidence for Pola‐R‐CHP in PLWH, as well as the potential risks and benefits, were discussed in detail, and the patient provided written informed consent for this treatment.

Pola‐R‐CHP was administered every 21 days; the first cycle was delivered in an inpatient setting, and cycles 2–6 were administered in the outpatient clinic (Figure 2(a)), at planned doses (Day 1: rituximab 375 mg/m^2^ and doxorubicin 50 mg/m^2^; Day 2: polatuzumab vedotin 1.8 mg/kg and cyclophosphamide 750 mg/m^2^; and prednisone 100 mg/day on Days 2–6), followed by two additional rituximab cycles (375 mg/m^2^), without dose reduction or treatment delay. Throughout treatment, BIC/FTC/TAF (50/200/25 mg; one tablet once daily) was continued without interruption or dose modification, as no clinically meaningful drug–drug interactions were anticipated between the ART regimen and Pola‐R‐CHP or concomitant prophylactic agents (Table 1) [11–21]. Supportive care included oral fluconazole, acyclovir, and sulfamethoxazole/trimethoprim, with pegfilgrastim administered once per cycle. The patient completed all planned cycles of Pola‐R‐CHP without dose reduction or unplanned hospitalization. No grade 3‐4 nonhematologic adverse events were observed (graded according to the National Cancer Institute Common Terminology Criteria for Adverse Events (CTCAE), Version 5.0 [22]); hematologic toxicity was manageable and did not lead to febrile neutropenia or serious infections. HIV‐1 RNA remained below 20 copies/mL during chemoimmunotherapy, and CD4+ T‐cell counts did not show a clinically relevant decline (Figure 2(a)).

**TABLE 1:** Drug–drug interaction assessment between BIC/FTC/TAF and concomitant medications.

Drug (role)	Primary metabolism/elimination	PK interaction with BIC/FTC/TAF
Bictegravir (BIC) (ART; INSTI)	Metabolized by CYP3A and UGT1A1; OCT2/MATE1 inhibitor; not a clinically relevant inhibitor of CYPs (incl. CYP3A) or UGT1A1 [11]	Not applicable (index regimen component; interaction assessment not required)
Emtricitabine (FTC) (ART; NRTI)	Not significantly metabolized; primarily renally eliminated (glomerular filtration + active tubular secretion) [11]	Not applicable (index regimen component; interaction assessment not required)
Tenofovir alafenamide (TAF) (ART; NRTI prodrug)	Prodrug; intracellularly hydrolyzed to tenofovir (major metabolite); hydrolysis mediated by cathepsin A (PBMCs/macrophages) and CES1 (hepatocytes); substrate of P‐gp and BCRP [11]	Not applicable (index regimen component; interaction assessment not required)
Polatuzumab vedotin (Pola‐R‐CHP component; ADC)	Expected antibody catabolism; unconjugated MMAE is a CYP3A4 substrate and P‐gp substrate [12]	No clinically significant interaction expected [13]
Rituximab (Pola‐R‐CHP component; anti‐CD20 mAb)	Proteolytic catabolism to peptides/amino acids (mAb); not metabolized by CYP enzymes [14]	No clinically significant interaction expected [13]
Cyclophosphamide (Pola‐R‐CHP component; alkylator)	Prodrug; activated by hepatic CYP enzymes (incl. CYP2A6, CYP2B6, CYP3A4/3A5, CYP2C9/2C18/2C19) [15]	No clinically significant interaction expected [13]
Doxorubicin (Pola‐R‐CHP component; anthracycline)	Clearance predominantly by metabolism and biliary excretion; P‐gp substrate; CYP3A4/CYP2D6 involvement [16]	No clinically significant interaction expected [13]
Prednisone (Pola‐R‐CHP component; glucocorticoid)	Converted to prednisolone; hepatic metabolism with urinary excretion of sulfate/glucuronide conjugates [17]	No clinically significant interaction expected [13]
Acyclovir (antiviral prophylaxis)	Primarily renal elimination; minimal metabolism (e.g., CMMG) [18]	No clinically significant interaction expected [13]
Sulfamethoxazole/trimethoprim (SMX/TMP) (PJP prophylaxis)	SMX: primarily renal excretion (free + metabolites); metabolism includes CYP2C9‐mediated N4‐hydroxylation; TMP: primarily renal elimination; OCT2 inhibitor [19]	Potential weak interaction (very low evidence); coadministration not studied; TMP may inhibit OCT2/MATE1 and could theoretically decrease FTC renal elimination; BIC unlikely to be clinically significant; no clinically significant interaction expected with tenofovir (from TAF) [13] ^∗^
Pegfilgrastim (G‐CSF support)	Large protein biologic; neutrophil count–dependent clearance (receptor binding–dependent, self‐regulating); nonlinear PK [20]	No clinically significant interaction expected [13] ^†^
Apremilast (plaque psoriasis therapy)	Extensively metabolized; primarily via CYP3A4 (minor CYP1A2/CYP2A6); also non‐CYP hydrolysis and glucuronidation [21]	No clinically significant interaction expected [13]

*Note:* BIC, bictegravir; FTC, emtricitabine; TAF, tenofovir alafenamide; ART, antiretroviral therapy; INSTI, integrase strand transfer inhibitor; PK, pharmacokinetic; CYP, cytochrome P450; CES1, carboxylesterase 1; P‐gp, P‐glycoprotein; CMMG, 9‐carboxymethoxymethylguanine; SMX, sulfamethoxazole; TMP, trimethoprim; MMAE, monomethyl auristatin E; mAb, monoclonal antibody.

Abbreviations: ADC, antibody–drug conjugate; BCRP, breast cancer resistance protein; G‐CSF, granulocyte colony‐stimulating factor; MATE1, multidrug and toxin extrusion protein 1; NRTI, nucleos(t)ide reverse transcriptase inhibitor; OCT2, organic cation transporter 2; PBMCs, peripheral blood mononuclear cells; PJP, *Pneumocystis jirovecii* pneumonia; UGT, uridine diphosphate‐glucuronosyltransferase.

^∗^Monitor serum creatinine; interpret mild increases as OCT2/MATE1‐mediated inhibition of tubular secretion (BIC/TMP) and may not reflect a true decline in glomerular filtration rate [11, 13, 19].

^†^Pegfilgrastim is not listed in the Liverpool HIV Drug Interactions database; this assessment is extrapolated from filgrastim (no interaction expected; very low evidence) [13].

End‐of‐treatment PET–CT scans demonstrated a marked metabolic response (Figures 2(e) and 2(f)). According to the Lugano criteria, the overall response was classified as a partial metabolic response because two small FDG‐avid foci persisted in the para‐aortic and right external iliac regions [8]; however, both residual lesions measured approximately 1 cm in diameter and had clearly decreased in size and FDG uptake compared with the baseline. This degree of tumor reduction was considered clinically sufficient, and no additional immediate systemic therapy was given. At the time of last follow‐up after the completion of Pola‐R‐CHP, the patient remained free from clinical or radiologic evidence of disease progression, with ongoing virologic suppression under BIC/FTC/TAF.

## 3. Discussion

This report describes the clinical course of an individual with HIV‐DLBCL who completed the planned treatment course—6 cycles of Pola‐R‐CHP followed by 2 additional rituximab cycles—while maintaining antiretroviral therapy with BIC/FTC/TAF. To our knowledge, this is the first reported case of Pola‐R‐CHP administered for HIV‐DLBCL. Two practical observations emerge: first, Pola‐R‐CHP appeared feasible without virologic breakthrough or unexpected toxicity in PLWH with preserved immune function; second, an outpatient‐based Pola‐R‐CHP strategy enabled treatment completion while accommodating patient priorities regarding social functioning and avoidance of prolonged hospitalization.

Evidence supporting Pola‐R‐CHP as a frontline option for DLBCL has been established in the general population [4], yet PLWH were often excluded from pivotal trials of novel cancer agents, leaving a persistent evidence gap in this population [5]. Notably, the POLARIX trial excluded individuals with HIV seropositivity [4]. Continued underrepresentation of PLWH in pivotal lymphoma trials may perpetuate uncertainty regarding the safety and effectiveness of contemporary regimens in this population, underscoring the need for real‐world evidence and deliberate inclusion of PLWH in prospective studies. In the present case, plasma HIV‐1 RNA remained suppressed (below 20 copies/mL) and CD4+ T‐cell counts were stable during chemoimmunotherapy, supporting the feasibility of administering a contemporary DLBCL regimen in the setting of immune reconstitution. A common concern in PLWH is the potential for pharmacokinetic drug–drug interactions between antiretroviral therapy, components of chemoimmunotherapy, and supportive medications. Based on known metabolic and transporter pathways, no clinically meaningful interactions were anticipated between BIC/FTC/TAF and the individual components of Pola‐R‐CHP or the concomitant prophylactic agents used during treatment; detailed pathway‐based assessments and supporting sources are summarized in Table 1 [11–21]. While a single case cannot establish safety, this experience supports our first practical observation, suggesting that Pola‐R‐CHP may be feasible in carefully selected PLWH without virologic breakthrough or unexpected toxicity. The planned treatment course was completed as intended.

Although cross‐trial comparisons are limited, prospective studies of rituximab‐containing CHOP‐ and EPOCH‐based regimens in HIV‐associated aggressive B‐cell lymphoma help interpret current regimen choices. In the randomized AMC 010 trial, adding rituximab to CHOP numerically increased complete response (CR) and unconfirmed complete response (CRu) rates (57.6% with R‐CHOP vs. 47% with CHOP), but this potential benefit was offset by higher treatment‐related infectious deaths (14% vs. 2%), particularly among patients with CD4 counts < 50 cells/μL [23]. In contrast, in a selected phase II cohort treated with R‐CHOP, Boué et al. reported a CR/CRu rate of 77% and an estimated 2‐year overall survival of 75% [24]. EPOCH‐based strategies have likewise shown high activity: in a prospective study of rituximab plus infusional EPOCH, concurrent administration achieved a higher CR rate than the sequential approach (73% vs. 55%) [25]. Notably, a DLBCL‐focused prospective study of short‐course EPOCH with dose‐dense rituximab reported durable long‐term disease control, with a 5‐year progression‐free survival of 84% [26]. Collectively, these prospective data help explain why EPOCH‐R is often considered in patients with HIV‐DLBCL; however, in real‐world practice, regimen selection may also be shaped by practical considerations related to treatment delivery and local care infrastructure.

The second observation relates to treatment delivery and implementation. In HIV‐DLBCL, some contemporary guidelines and reviews favor EPOCH‐R in selected patients [1, 3], while R‐CHOP is also commonly used in routine practice [2]. However, high‐quality head‐to‐head comparisons in PLWH remain limited [1–3]. Although outpatient EPOCH‐based programs have been reported, including a single‐center inpatient/outpatient program in the United States and home‐based delivery in Denmark, implementation remains infrastructure dependent, typically requiring continuous infusion, central venous access, portable infusion pumps, and dedicated ambulatory support [27, 28]. In real‐world care, outpatient‐compatible regimens may be considered for selected individuals when prolonged hospitalization is undesirable, following shared decision‐making and careful assessment of immune status and supportive care needs. In contrast, Pola‐R‐CHP is generally compatible with outpatient delivery. The patient reported that after receiving the first cycle in an inpatient setting, subsequent outpatient cycles helped him maintain his social activities and avoid prolonged hospitalization, with less disruption to his routine. Thus, in the present case, Pola‐R‐CHP represented a practical outpatient‐compatible option that was better aligned with the local care environment and the patient’s preference to avoid prolonged hospitalization. From an implementation perspective, aligning frontline lymphoma therapy with patient preferences may be particularly relevant in PLWH, for whom maintaining continuity of HIV care and adherence to antiretroviral therapy are integral to outcomes.

Several limitations should be acknowledged in this report. This is a single case, and its generalizability is constrained by patient selection: immune function was preserved and plasma HIV‐1 RNA was suppressed prior to lymphoma therapy, conditions that may not apply to all PLWH with DLBCL. Follow‐up duration was limited, rare toxicities or delayed effects on HIV‐related parameters may not have been captured, and comparative effectiveness versus EPOCH‐R or other regimens cannot be inferred.

## 4. Conclusion

Outpatient‐delivered Pola‐R‐CHP for HIV‐DLBCL was feasible in PLWH with durable virologic suppression and immune reconstitution receiving BIC/FTC/TAF, without virologic breakthrough, unexpected toxicity, or serious infections. This experience suggests that Pola‐R‐CHP may be a practical option for carefully selected patients with HIV‐DLBCL when delivery of EPOCH‐R is logistically challenging or inconsistent with patient preferences, provided that supportive care and drug–drug interaction assessment are rigorously implemented. Prospective inclusion of PLWH in polatuzumab‐containing frontline studies is warranted to better define safety and effectiveness.

## Author Contributions

Yoshikazu Ikoma, Masashi Ishihara, Hitomi Sugiyama, Daisuke Okamoto, Naoki Hayase, Kimihiro Yamaguchi, Takuro Matsumoto, Nobuyuki Tetsuka, Nobuhiko Nakamura, Hiroshi Nakamura, Nobuhiro Kanemura, Masahito Shimizu, and Hisashi Tsurumi contributed to the conception of the case. Yoshikazu Ikoma and Masashi Ishihara collected the clinical data. Yoshikazu Ikoma and Hisashi Tsurumi drafted the manuscript. Nobuhiro Kanemura, Masahito Shimizu, and Hisashi Tsurumi critically revised the manuscript.

## Funding

No funding was received for this study.

## Disclosure

All authors have approved the final version of the manuscript and are accountable for all aspects of the work.

## Ethics Statement

This report was conducted in accordance with the Declaration of Helsinki. Institutional review board approval was not required according to local regulations because this report describes a single anonymized patient and involved no procedures beyond routine clinical practice.

## Consent

Written informed consent for publication of this report and all accompanying images was obtained from the patient.

## Conflicts of Interest

Yoshikazu Ikoma has received honoraria from AbbVie GK, Astellas Pharma Inc., Meiji Seika Pharma Co., Ltd., Nippon Shinyaku Co., Ltd., Kyowa Kirin Co., Ltd., and Chugai Pharmaceutical Co., Ltd.

Nobuhiko Nakamura has received honoraria from Chugai Pharmaceutical Co., Ltd., Kyowa Kirin Co., Ltd., AbbVie GK, Janssen Pharmaceutical K.K., Sanofi, Novartis, MSD K.K., Sumitomo Pharma Co., Ltd., Nippon Kayaku Co., Ltd., Nippon Shinyaku Co., Ltd., Meiji Seika Pharma Co., Ltd., and AstraZeneca K.K.

Masashi Ishihara, Nobuyuki Tetsuka, and Hisashi Tsurumi have received honoraria from Gilead Sciences, Inc.

Nobuyuki Tetsuka has also received honoraria from Sanofi, Shionogi & Co., Ltd., Beckman Coulter, Inc., and Becton, Dickinson and Company.

The remaining authors declare no conflicts of interest.

## Data Availability

The data that support the findings of this case report are available on request from the corresponding author. The data are not publicly available due to privacy or ethical restrictions.
